# Active Brain-Computer Interfacing for Healthy Users

**DOI:** 10.3389/fnins.2022.859887

**Published:** 2022-04-25

**Authors:** Sergei L. Shishkin

**Affiliations:** MEG Center, Moscow State University of Psychology and Education, Moscow, Russia

**Keywords:** brain-computer interfaces, active BCI, human-computer interaction, human-machine interfaces, healthy users

## Introduction

Brain-computer interface (BCI) research and development continues to grow. In particular, BCI patent applications have been increasing exponentially in a few recent years (Greenberg et al., 2021). The situation is, however, different for different kinds of BCI: *invasive* and *non-invasive, active* and *passive*, especially regarding possible use by healthy users. *Invasive* BCIs provide best performance, and even may provide access to early stages of motor decision formation, enabling faster interaction compared to usual input devices (Mirabella and Lebedev, 2017), but they are associated with high risk and cost, and will unlikely be available for healthy users in near future. Existing *non-invasive* BCIs have low bandwidth, speed, and accuracy, and this is why only *passive*, not active BCIs have been considered as a prospective technology for healthy users in the roadmap of brain/neural-computer interaction (BNCI Horizon 2020, 2015; Brunner et al., 2015). Passive BCIs are those that use “brain activity arising without the purpose of voluntary control” (Zander and Kothe, 2011). As they do not claim the user's attention, their low speed of interaction can be acceptable (Current Research in Neuroadaptive Technology, 2021).

In contrast, a user of an *active* BCI controls an application explicitly, via conscious control of his or her brain activity (Zander and Kothe, 2011)^1^. These BCIs have to compete with the manual input devices (keyboard, mouse, touchscreen) and emerging touchless alternatives (voice-, gesture- and gaze-based), as playing the same role in human-computer interaction (HCI) (Lance et al., 2012; van Erp et al., 2012). Although some attempts were announced to dramatically improve performance of the non-invasive BCIs by advancing brain sensor technology (most noticeably, Facebook's plans to enable fast text input “directly from your brain”—Constine, 2017), the electroencephalography (EEG) remains the only widely used technology and performance is still below from what is provided by electromechanical input devices. For example, the best reported average time of activation of a non-invasive asynchronous “brain switch” (a BCI requiring low false positive rate but enabling detection of only one discrete command) is about 1.5 s (Zheng et al., 2022). Moreover, while some non-medical active BCIs use well-established non-invasive BCI paradigms—the motor imagery BCI, the P300 BCI, the steady-state visual evoked potential (SSVEP) BCI and the code-modulated visual evoked potential (c-VEP) BCI—many projects rely on even less precise control based on learned changing EEG rhythms (Nijholt, 2019; Prpa and Pasquier, 2019; Vasiljevic and de Miranda, 2020). Due to low performance, active BCIs are still affordable mainly for people who cannot use other input, such as paralyzed individuals.

Nevertheless, attempts to develop active BCIs for healthy people continue. In this Opinion, I briefly overview the application areas for which they are currently developed, then try to figure out what motivates these attempts, and what is the near perspective.

## Applications

What types of non-medical applications of active BCIs have been developed and studied in recent years? In my view, most of them fall into one of the several groups:

*1. Games*—BCI gaming remains the most studied application of active BCI for healthy users (Vasiljevic and de Miranda, 2020). In this application, input imprecision inherent to non-invasive BCIs is not always as critical as in most real-life applications, and even can serve as a part of intentionally constructed uncertainty within the gameplay (Nijholt et al., 2009). Commercial EEG devices for gaming have been produced for more than 10 years, and games developed for them are becoming increasingly user-friendly (Vasiljevic and de Miranda, 2020). Both active and passive BCIs are studied as means to interact with games, but both are still far from becoming a widely accepted input for games, which is partly due to low performance. Low popularity of the BCI games in the gamer community can also be related to insufficient attention to studying interaction in BCI games, developing relevant game design and software and hardware solutions (Vasiljevic and de Miranda, 2020; Cattan, 2021).

*2. Art*—Another BCI application for healthy users is the use of BCI by enthusiast artists in performances and creating pieces of art, i.e., “brain art” (Nijholt, 2019) or “BCI art” (Prpa and Pasquier, 2019). These projects are very diverse (Brain Art, 2019; Bernal et al., 2021), but, unfortunately, rarely documented in the scientific literature (Prpa and Pasquier, 2019; Friedman, 2020). Of 61 BCI art projects surveyed by Prpa and Pasquier (2019), mostly described in non-science sources such as YouTube videos, 18 used active or reactive control (Table 3.4 in Prpa and Pasquier, 2019). For brain art, like for the BCI games, robustness and efficiency may be considered less important than experience (Nijholt et al., 2022).

*3. Autonomous-driving vehicles*—BCI control of autonomous vehicles is increasingly considered for healthy users (Rehman et al., 2018; Chai et al., 2021; Hekmatmanesh et al., 2021). Such BCI presented by Mercedes-Benz in their concept car (Rosso, 2021) enabled “selecting the navigation destination by thought control, switching the ambient light in the interior or changing the radio station” (Mercedes-Benz VISION AVTR, 2021).

*4. Augmented and virtual reality (AR/VR)*—While these technologies are quickly improving, input in AR/VR is still far from perfect. Therefore, active BCIs have some chances to compete, either as a general-purpose AR/VR input mean or in connection with BCI games and BCI art (Putze, 2019; Cattan et al., 2020; Paszkiel, 2020; Wen et al., 2021). Noticeably, NextMind, the company that provided their BCI for the above-mentioned Mercedes car (Rosso, 2021), was recently purchased by an AR developer (Heath, 2022).

Attempts were also made to develop BCIs which could be used to enable additional input when the two arms are busy (“third arm”; Penaloza and Nishio, 2018), or even replacing normal input devices in some tasks by providing more effortless and fluent control (“wish mouse,” Shishkin et al., 2016). In these areas BCI performance remains significantly lower than what is acceptable for practical applications.

## Motivations

Why do some BCI developers expect that healthy users would prefer BCIs over other, more accurate, faster, and robust input technologies?

*1. Practical reasons*—AR/VR and, less obviously, autonomous-driving cars are special cases where traditional input means do not fit the technology well. Here, BCIs compete with emerging control approaches based on the movements of the head, body, hands (gestures), and gaze, each of which has its own shortcomings. Moreover, if a user wears a head-mounted display, adding BCI control to it is not necessarily associated with significant inflation of the price and increased inconvenience. In an autonomous-driving car, the increase of price would be even less noticeable; in this case, there is a range of tasks where response time and accuracy are not critical issues as well (see above the Mercedes example). However, in almost all applications productivity and efficiency are not what non-invasive BCIs are valued for (I refrain here from discussing neurofeedback-based training, which is typically based on technologies somewhat different from BCI—the only exception, to my knowledge, is Arvaneh et al., 2019).

*2. Experience*—In HCI, not only productivity and efficiency are valuable, but also, increasingly, various aspects of interaction experience, such as “affect, comfort, family, community, or playfulness,” where BCI technologies have certain advantages (Bernal et al., 2021; Nijholt et al., 2022). In some cases, BCI-based interaction brings highly paradoxical experience: for example, the long-known feature of control based on alpha rhythm is “the more you try, the less likely is to succeed” (Lucier and Simon, 1980, cited by Prpa and Pasquier, 2019, p. 102). User experience is especially important for BCI art (Nijholt, 2019; Nijholt et al., 2022) but also for BCI games and AR/VR (Vasiljevic and de Miranda, 2020; Cattan, 2021; Nijholt et al., 2022), and even for autonomous driving (where the goal for a BCI is “to further enhance driving comfort in the future” and to open up “revolutionary possibilities for intuitive interaction with the vehicle,” Mercedes-Benz VISION AVTR, 2021).

Unique BCI experience in BCI art and in some BCI games can be partly associated with one interesting feature of BCI-based control, not found in computer inputs which exclude passive interaction: *an active BCI makes possible passive BCI control, and vice versa*. As Anton Nijholt explained: “Obviously, when a subject is told to wear a BCI cap he or she can become aware and learn how changes are related to a mental state and can turn passive BCI into active BCI by producing different mental states. A subject's active and reactive BCI performance can be dependent on his or her mental state” (Nijholt, 2019, p. 6). It is tempting to hypothesize that this “fuzziness” of the conscious control may open the door for the user's unconsciousness to cause desirable but suppressed actions. This can help artists to express something that is difficult to express in other ways, and possibly may lead to unusual engaging experiences in games. To my knowledge, such “fuzziness” has never been addressed in experimental research.

Moreover, the experience of healthy users of active BCI control was very little studied so far (Vasiljevic and de Miranda, 2020; Cattan, 2021). The most systematic study, to my knowledge, was conducted by Schmid and Jox (2021), who engaged (apart from professional BCI researchers and developers) only three participants with regular BCI use experience (BCI gamers).

## Perspectives

As the previous two sections suggest, the development of active BCIs for healthy users continued in recent years, but the focus was on applications for which user experience was more valuable than productivity and efficiency. More attention of researchers and developers to experience-related issues can therefore help strongly improve affordability of these BCIs in the near future (Vasiljevic and de Miranda, 2020; Cattan, 2021).

Even though the unique experience of interaction mediated by active BCIs provides certain advantages in their competition with traditional input means, improvement of BCI performance is still highly desirable. One possible way is the use of deep neural networks as BCI classifiers (Craik et al., 2019; Roy et al., 2019). However, such classifiers often have many parameters, and therefore rarely can be well-trained on single-session data. The current trend of the increased availability of large datasets, on which more advanced classifiers can be learned, therefore may make possible significant improvement of performance. Further development of transfer learning (e.g., Zanini et al., 2017; Fahimi et al., 2019; Dehghani et al., 2021) and more recent meta-learning (Li et al., 2021; Bhosale et al., 2022; Wei et al., 2022) approaches make possible applying a classifier trained on large multisubject datasets to the data from new users. Additional opportunities can be found in combining different BCI modalities and creating hybrid systems based on joint use of a BCI and other input devices (Wen et al., 2021).

Improved performance may make feasible modifications of existing BCI paradigms that provide more intensive experience. In BCI games, for example, better classification may help to turn the P300 paradigm into single-trial (Finke et al., 2009; Ganin et al., 2013) and single-stimulus (Fedorova et al., 2014) modifications, enabling higher integration with gameplay and higher immersion (Kaplan et al., 2013); “quasi-movement” paradigm (Nikulin et al., 2008) may offer easier training and, possibly, more intensive experience than traditional motor imagery BCI.

If passive BCIs will become widely used by healthy users, their hardware could be used for active BCIs. Similarly, wide use of gaze-based control by healthy users may make hybrid interfaces using gaze and EEG also more affordable.

In summary, while non-invasive active BCIs for healthy users are not currently a mature technology, further efforts of researchers and developers may soon lead to creation of affordable products.

## Author Contributions

The author confirms being the sole contributor of this work and has approved it for publication.

## Funding

This work was supported by the Russian Science Foundation, grant 22-29-01361.

## Conflict of Interest

The author declares that the research was conducted in the absence of any commercial or financial relationships that could be construed as a potential conflict of interest.

## Publisher's Note

All claims expressed in this article are solely those of the authors and do not necessarily represent those of their affiliated organizations, or those of the publisher, the editors and the reviewers. Any product that may be evaluated in this article, or claim that may be made by its manufacturer, is not guaranteed or endorsed by the publisher.

## Footnotes

^1^Zander and Kothe (2011) suggested a distinction between *active* and *reactive* BCIs, the latter depending on “brain activity arising in reaction to external stimulation, which is indirectly modulated by the user”. Here, I use the term “active BCI” for both these BCIs, as they both enable explicit, intentional control, with active role of the user.
